# *Mycobacterium tuberculosis* Rv3406 Is a Type II Alkyl Sulfatase Capable of Sulfate Scavenging

**DOI:** 10.1371/journal.pone.0065080

**Published:** 2013-06-06

**Authors:** Kimberly M. Sogi, Zev J. Gartner, Mark A. Breidenbach, Mason J. Appel, Michael W. Schelle, Carolyn R. Bertozzi

**Affiliations:** 1 Department of Chemistry, University of California, Berkeley, California, United States of America; 2 Department of Molecular and Cell Biology, University of California, Berkeley, California, United States of America; 3 Howard Hughes Medical Institute, University of California, Berkeley, California, United States of America; 4 Department of Pharmaceutical Chemistry, University of California San Francisco, San Francisco, California, United States of America; Institut de Pharmacologie et de Biologie Structurale, France

## Abstract

The genome of *Mycobacterium tuberculosis* (Mtb) encodes nine putative sulfatases, none of which have a known function or substrate. Here, we characterize Mtb’s single putative type II sulfatase, Rv3406, as a non-heme iron (II) and α-ketoglutarate-dependent dioxygenase that catalyzes the oxidation and subsequent cleavage of alkyl sulfate esters. Rv3406 was identified based on its homology to the alkyl sulfatase AtsK from *Pseudomonas putida.* Using an *in vitro* biochemical assay, we confirmed that Rv3406 is a sulfatase with a preference for alkyl sulfate substrates similar to those processed by AtsK. We determined the crystal structure of the *apo* Rv3406 sulfatase at 2.5 Å. The active site residues of Rv3406 and AtsK are essentially superimposable, suggesting that the two sulfatases share the same catalytic mechanism. Finally, we generated an Rv3406 mutant (Δ*rv3406*) in Mtb to study the sulfatase’s role in sulfate scavenging. The Δ*rv3406* strain did not replicate in minimal media with 2-ethyl hexyl sulfate as the sole sulfur source, in contrast to wild type Mtb or the complemented strain. We conclude that Rv3406 is an iron and α-ketoglutarate-dependent sulfate ester dioxygenase that has unique substrate specificity that is likely distinct from other Mtb sulfatases.

## Introduction

**Figure 1:**
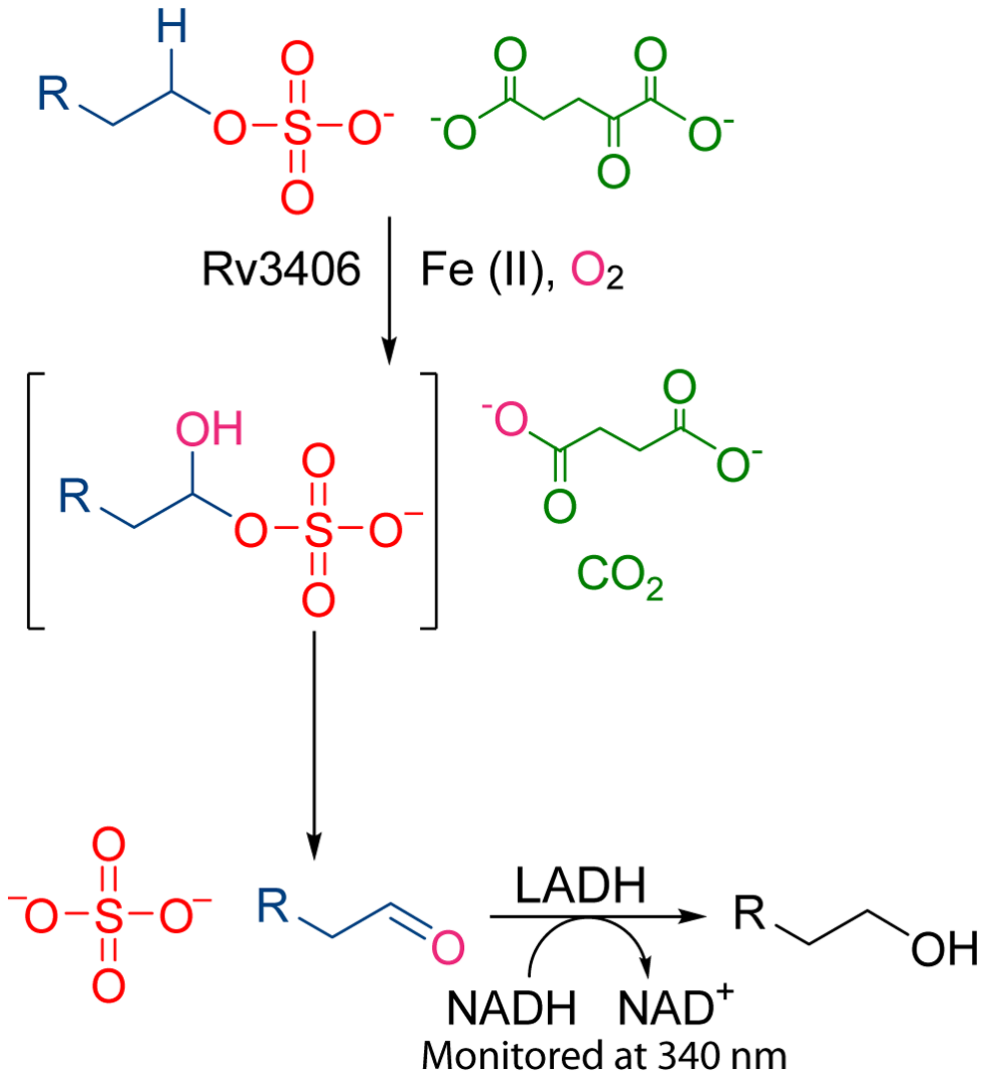
The reaction catalyzed by Rv3406. The alpha carbon of an alkyl sulfate is oxidized by Rv3406 in the presence of α-ketoglutarate and spontaneously collapses to an aldehyde and sulfate, liberating CO_2_ and succinate. The product formation was monitored using a coupled assay using LADH to reduce the aldehyde in a NADH dependent manner.

Sulfatases catalyze the cleavage of sulfate esters and are involved in diverse biological processes. Desulfation of biomolecules has been found to regulate cell signaling, hormone activity, and tissue remodeling in animals, and may also be important for sulfate scavenging and metabolism [1], [2], [3], [4], [5]. The roles of sulfatases in prokaryotes are less well defined, though most characterized bacterial genomes are predicted to encode at least one such enzyme. To date, three classes of sulfatases have been identified. The type I sulfatase family requires posttranslational modification of a cysteine or serine residue within a conserved consensus motif to a catalytically essential formylglycine residue [6], [7], [8], [9]. The human genome encodes only type I sulfatases, which cleave the RO–SO_3_^–^ bond and consume one equivalent of water in the process. Two additional types of sulfatases have been identified in prokaryotic genomes. Type III sulfatases hydrolyze the same bond as do the type I enzymes, employing a Zn^2+^ cofactor to activate a nucleophilic water molecule using a mechanism related to that of some metalloproteases [10]. By contrast, type II sulfatases are non-heme iron-dependent dioxygenase that oxidize the C–H bond alpha to the sulfate ester using α-ketoglutarate (αKG) and oxygen as substrates (Fig. 1). The resulting hemiacetal sulfate ester collapses, liberating inorganic sulfate and an alkyl aldehyde, as well as water, carbon dioxide, and succinic acid byproducts [11] (Fig. 1). Thus, type II sulfatases are unique within the sulfatase superfamily in that they cleave the R–OSO_3_^–^ bond.

The environmental niche occupied by a particular microorganism can be suggestive of a role for the sulfatases encoded by its genome. For example, the vast majority of sulfate found in soil is trapped in the form of organic sulfate esters [12]. Not surprisingly, soil bacteria, such as *Pseudomonas putida*, encode a variety of sulfatases that are upregulated under conditions of sulfate starvation, suggesting a role in sulfate scavenging [13]. These sulfatases are part of a larger transcriptional response that includes multiple enzymes necessary for the uptake and reduction of sulfate into reduced sulfur-containing metabolites (e.g., cysteine, methionine, coenzyme A, etc). The genome of the marine bacterium *Pirellula sp.* contains an incredible 110 sulfatases; a role for these enzymes in sulfate scavenging is unlikely due to the ocean’s abundance of free inorganic sulfate [14]. Instead, *Pirellula sp*.’s sulfatases are believed to be involved in the breakdown of heterogeneously sulfated polysaccharides, a major component of phytodetrital macroaggregates (“marine snow”) and an abundant source of carbon for these microorganisms [14].

In the case of pathogenic bacteria, sulfatase enzymes are known to play additional roles in the colonization of tissues and the maintenance of infection. Several reports link general sulfatase activity to pathogenicity across a variety of microorganisms involved in human diseases [1], [15]. These include infection of the lung [16], [17], stomach [18], lower GI tract [19], and central nervous system [20]. While the specific functions of the sulfatase enzymes in pathogenic bacteria have not been well defined, they may participate in sulfate scavenging or the breakdown of sulfated biomolecules for use as a source of carbon.

The genome of *Mycobacterium tuberculosis* (Mtb), the causative agent of the disease tuberculosis, encodes a total of nine putative sulfatases: one type II sulfatase (Rv3406), three type III sulfatases (Rv2407, Rv3796, and Rv3762c), and five type I sulfatases (AtsA, AtsB, AtsD, AtsF, AtsG). The sulfatases’ substrates are not known and their roles in pathogenesis have not been addressed [21]. More generally, however, sulfate metabolism has been shown to be critical for Mtb’s virulence in mouse infection models and several enzymes involved in sulfur assimilation have been named as promising drug targets [22], [23].

Here we characterized the single putative type II sulfatase from Mtb, Rv3406, which is the most highly conserved sulfatase among mycobacterial species. The *rv3406* gene product was originally annotated as a taurine dioxygenase-like enzyme [24]; this class of enzymes uses the same cosubstrates as type II sulfatases but cleave a sulfonate R–SO_3_^–^ bond (releasing sulfite) rather than a C–O bond. Our bioinformatics analysis suggested that Rv3406 is more closely related to the previously characterized alkyl sulfatase AtsK from *Pseudomonas putida* strain S313 [25]. AtsK, like all αKG and non-heme iron-dependent dioxygenases, possesses an essential iron binding motif H-X-D/E-X_n_-H, where X is any amino acid, as well as a conserved arginine involved in αKG binding [26]. Rv3406 shares 54% sequence identity and 66% similarity with *P. putida* AtsK, including both the iron binding motif (His^99^, Asp^101^, His^165^) and the arginine residue (Arg^267^), suggesting it possesses similar enzymatic activity.

To determine whether Rv3406 functions as an AtsK-like alkyl sulfatase, we expressed the recombinant protein in *E. coli* and tested its activity on a panel of sulfated substrates. We found medium-chain alkyl sulfates to be good substrates for Rv3406, particularly 2-ethylhexyl sulfate (2-EHS). Further, we determined the crystal structure of *apo* Rv3406 at 2.5 Å, thereby confirming that its overall fold and active site are structurally similar to *P. putida* AtsK. Finally, we demonstrated that Rv3406 is essential for Mtb growth with 2-EHS as the sole sulfur source, indicating that Rv3406 has alkyl sulfatase activity in live Mtb and can aid in sulfate scavenging.

## Materials and Methods

### Bacterial Strains and Growth Media

Growth media 7H9 and 7H11 and OADC supplement were obtained from BD Biosciences. Cloning and plasmid propagation were performed in *Escherichia coli* DH5α and XL-1Blue strains. All mutants were made in Mtb Erdman strain (ATCC 35801). The Mtb growth medium was 7H9 (liquid) or 7H10/7H11 (solid) with 0.5% glycerol, 0.05% Tween-80, and 10% OADC. For sulfate starvation, Sauton media [27] was used where MgSO_4_ and ZnSO_4_ were replaced with molar equivalents of MgCl_2_ and ZnCl_2_. Antibiotics carbenicillin and kanamycin were obtained from Sigma and hygromycin from Invitrogen as a 50 mg/mL solution in PBS. For selective media, antibiotic concentrations were 100 µg/mL carbenicillin, 50 µg/mL kanamycin or 100 µg/mL hygromycin for *E. coli* and 20 µg/mL kanamycin or 50 µg/mL hygromycin for Mtb.

### Cloning, Expression and Protein Purification

*Pfu* DNA polymerase was from Stratagene (La Jolla, CA). Oligonucleotides were from Elim Biopharmaceuticals, Inc. (Hayward, CA). Restriction enzymes were from New England Biolabs (Ipswich, MA). Qiagen (Valencia, CA) kits were used for plasmid DNA purification and the extraction of DNA from agarose gels. T4 DNA ligase was purchased from Invitrogen. DNA sequencing was performed by Elim Biopharmaceuticals, Inc.

DNA encoding full-length Rv3406 protein (residues 2–295) was inserted into the pET-28b expression vector (Novagen) between NdeI and XhoI restriction sites in frame with the vector’s N-terminal, thrombin cleavable hexa-histidine affinity tag. The expression vector was transformed into chemically competent BL21 (DE3) *E. coli* (Stratagene) and a single colony was used to inoculate 2 L cultures of LB with 100 µg/mL kanamycin. Cultures were incubated at 37°C with shaking until mid-log phase (OD_600_ = 0.7) at which point the temperature was decreased to 18°C and protein expression was induced with 250 µM isopropyl β-D-1-thiogalactopyranoside. Induced cultures were incubated overnight before harvest by centrifugation. Cell pellets were flash-frozen in liquid nitrogen and stored at −80°C until ready for use.

Frozen cell pellets were resuspended in a 1∶5 (w/v) ratio of lysis buffer (50 mM HEPES, pH 7.5, 150 mM NaCl, 0.5 mM DTT). Protease inhibitors added to the lysis buffer included 0.5 mM phenylmethylsulfonyl fluoride and EDTA-free Complete Protease Inhibitor Cocktail Tablets (Roche). Cells were lysed via three passes through an Emulsiflex-C3 homogenizer (Avestin) at approximately 15,000 psi. Insoluble debris was removed by centrifugation in a SS-34 rotor (Sorvall) at 19,500 rpm for 45 min at 4°C. Clarified lysate was incubated with Ni-NTA agarose resin (Qiagen) in batch for 4 h at 4°C; resin was subsequently washed with 10 column volumes of lysis buffer. Bound protein was eluted with 25 mL of elution buffer (50 mM HEPES pH 7.5, 300 mM imidazole, 150 mM NaCl, 0.5 mM DTT). Proteins in the Ni-NTA eluate were exchanged into low-salt buffer (20 mM Tris pH 7.4, 1 mM DTT) and Rv3406 was subsequently purified to near-homogeneity by anion-exchange chromatography with MonoQ resin (GE healthcare). Rv3406 was used with the intact His_6_-tag in kinetic assays. For crystallography, the affinity tag was removed by adding bovine α-thrombin (Haematologic Technologies) to Rv3406 at a 1∶2000 ratio (w/w) and dialyzing overnight at 4°C against 20 mM Tris pH 7.5, 150 mM NaCl, and 1 mM DTT. The thrombin-cleaved product, which retained three N-terminal residues (gly-ser-his) as a cloning artifact, was further purified by size-exclusion chromatography with Sephacryl S-300 resin (GE Healthcare). Purity of Rv3406 was assessed by SDS-PAGE and was quantified by UV/vis spectroscopy at 280 nm based on its theoretical extinction coefficient in denaturing conditions.

### Biochemical Characterization of Rv3406

All chemicals were purchased from Sigma, Spectrum (New Brunswick, NJ), or Fluka (St. Louis, MO) and used without further purification. Unless stated in the text, all enzymatic assays were carried out following the standard conditions as previously described [25]. Briefly, all biochemical reactions were performed in 200 µL and carried out at 25°C in glass cuvettes. Reactions contained 40 mM Tris acetate buffer, pH 7.5, 50 mM NaCl, 0.2% triton, 100 µM iron (II) chloride, 1 mM αKG, 2 µM ascorbate, 175 µM NADH, and 1 unit of horse liver alcohol dehydrogenase (LADH). The substrates were tested at 50 µM except where noted. Reactions were started by addition of 0.25 µL of Rv3406 (stock 20 mg/mL) and monitored for NADH depletion at 340 nm. UV/Vis/NIR spectra were acquired on a CARY 100 Bio UV-Visible Spectrophotometer with a range of 200–900 nm. Reactions lacking Rv3406 or LADH were used as negative controls. For the determination of iron and ascorbate dependence, iron concentrations ranged from 0.012 µM to 12.5 µM and ascorbate concentrations were varied from 400 nM to 10 mM with all other components held constant.

### Protein Crystallization and Structure Determination

Purified Rv3406 protein was concentrated to 14 mg/mL in apo crystallization buffer (10 mM Tris pH 7.5, 150 mM NaCl, and 1 mM DTT). Crystals of Rv3406 were grown by hanging-drop vapor diffusion over a 0.5 mL reservoir of 22% (w/v) PEG 2000, 300 mM Mg(NO_3_)_2_, 100 mM Tris pH 8.0, and 2% 2-methyl-2,4-pentanediol (MPD) incubated at 18°C over a period of 2–3 days. Octahedral crystals of high aesthetic quality were harvested when they had grown to approximately 100–200 µm length on each edge. Individual crystals were harvested into artificial mother liquor (AML; 25% PEG 2000, 100 mM Tris pH 8.0, 2% MPD) and were stable for months at room temperature. Prior to data collection, crystals were flash-frozen in liquid N_2_ after brief exposure to cryoprotectant consisting of AML supplemented with 20% MPD. Single crystal diffraction data were collected on a Quantum-315 CCD detector (Area Detector Systems) at beamline 8.2.2 at the Advanced Light Source, Lawrence Berkeley National Lab. Reflections were observable to approximately d_min_ = 2.5 Å. The sample was mounted on a single-axis goniostat, and 1° oscillations spanning a 140° range were collected at 11,500 eV. Initial phases were determined by molecular replacement (MR) using Phaser [28]. The MR search probe was a monomeric polyalanine scaffold derived from coordinates for the *P. putida* alkyl sulfatase AtsK from PDB entry 1OIH [11]. Four monomers were placed in the asymmetric unit, consistent with a V_M_ of 2.2 Å^3^ and 45% solvent content. The final model was built via iterative cycles of model editing using Coot [29] and maximum likelihood refinement with PHENIX [30]. The progress of model refinement was monitored by cross-validation using R_free_ [31] which was computed from a randomly assigned test set comprising 5% of the data. Non-crystallographic symmetry restrains were not used. One NO_3_^2−^ ion was modeled into trigonal planar electron density sandwiched between symmetry-related copies of the ASU. Model quality was evaluated using MolProbity [32]. Disordered regions varied slightly amongst the four monomers in the ASU, and included between 6–7 residues at each N-terminus and between 1–10 residues at each C-terminus. Additionally, each monomer had two internal disordered loops approximately spanning gly69–lys92 (DL1) and tyr155–pro177 (DL2). Data, refinement, and model quality indicators are summarized in Table S1. Images and structural alignments were generated with PyMol (Schrödinger, LLC). The coordinates have been deposited in the Protein Data Bank (PDB) entry 4FFA.

### Construction of Mtb Δrv3406 Knockout Mutant

The Δ*rv3406* mutant strain was created by homologous recombination as previously described [33]. Briefly, specialized transduction phage phMWS130 was incubated with concentrated Mtb strain Erdman cells for 4 h at 39°C. Cells were then plated on 7H10 plates containing hygromycin. Colonies were picked and screened for the disruption by PCR, which confirmed the replacement of 592 bp of *rv3406* (encoding amino acids 57 through 254) with a hygromycin resistance cassette. The Δ*rv3406*::*rv3406* complementation strain was created by cloning the *rv3406* gene from Mtb strain Erdman into the mycobacterial expression vector pMV306, a derivative of the pMV361 vector [34] with a multiple cloning site in place of the expression cassette and containing the glutamine synthase promoter. The resulting plasmid was electroporated into Δ*rv3406* and transformants were selected on 7H11 kanamycin-containing plates.

### Growth Measurements of Mtb Strains during Sulfate Starvation

Mtb strains were grown for 3–5 days to late-log phase in 7H9 media. Cultures were washed in sulfate-free Sauton media and grown for two days. The Mtb strains were diluted to OD_600_ 0.1 in sulfate free Sauton media and supplemented with (a/d) water, (b) 1 mM 2-EHS, (c) 0.5 mM 2-EHS and 0.5 mM sodium sulfate, (e) 1 mM *n*-heptyl sulfate, or (f) 0.02% SDS (w/v). Unfortunately, attempts to monitor growth by optical density or colony forming units were complicated by the observation that all strains aggregated rapidly under the growth conditions. Instead, growth was monitored by intracellular ATP using BacTiter-Glo Microbial Cell Viability Kit (Promega) [35], [36]. One milliliter aliquots of cultures were taken on day 5 and immediately heat inactivated. Samples were stored at −20°C until analysis. Twenty-five microliters of cell lysates were transferred into white 96 well plates, mixed with an equal volume of the BacTiter–Glo reagent and incubated for 5 min in the dark. Luminescence was read on a luminometer (Gemini XPS fluorescence microplate reader, Molecular Devices Corporation) and was expressed as relative luminescence units (RLU). ATP standards ranging from 0.1 to 1 µM were included in each plate of the experiments as internal controls.

## Results

### Biochemical Characterization of Rv3406

**Figure 2:**
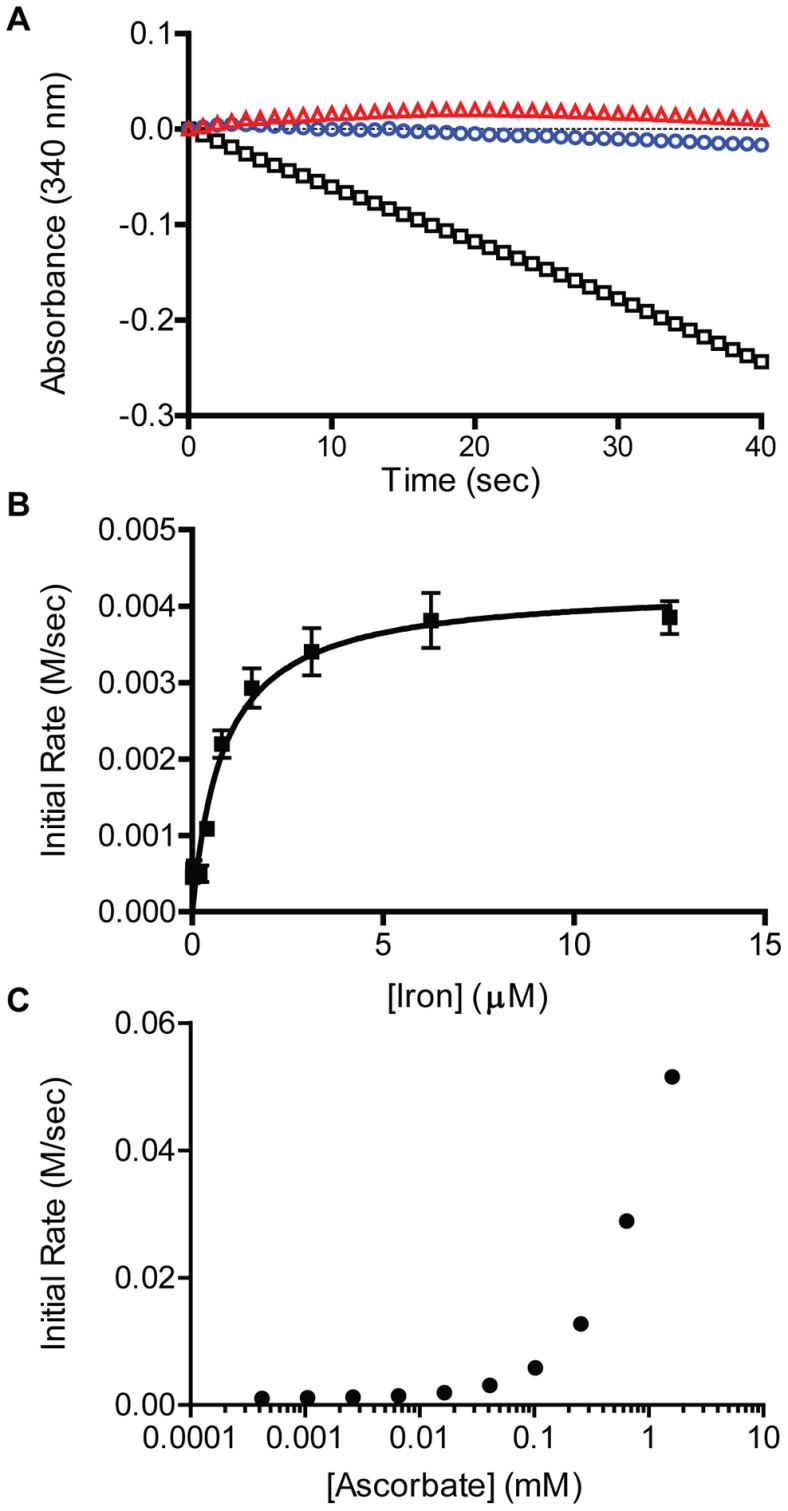
Biochemical characterization of Rv3406. (A) Rv3406 is an αKG and ascorbate dependent sulfatase. Black squares are the complete assay with Rv3406, 2-EHS, αKG, ascorbate and iron. Red triangles are without 2-EHS. Blue circles are without αKG. (B) Rv3406 is an iron dependent enzyme. (C) The rate of Rv3406 accelerates with the addition of ascorbate up to 1 mM. Rv3406 enzyme concentration was between 0.5 and 0.75 µM for all experiments.

To test for alkyl sulfatase activity *in vitro*, we used a NADH/liver alcohol dehydrogenase (LADH) coupled assay to detect the expected aldehyde product [25]. We tested a panel of alkyl sulfate esters (Table 1) and found optimal activity on medium-chain substrates, particularly 2-EHS (Fig. 2A). *n*-Hexyl and *n*-heptyl sulfate showed moderate substrate activity, and *n*-pentyl sulfate was inefficiently desulfated (Fig. S1A). As a point of comparison, we also tested AtsK and found 2-EHS was its preferred substrate as well. As expected, Rv3406 was inactive on the aryl sulfate 4-methylumbelliferyl sulfate (4-MUS) [21], a fluorogenic substrate often used in the study of type I sulfatases that lacks the requisite C-H bond that is cleaved in the type II sulfatase reaction. Notably, Rv3406 was also inactive on taurine, further strengthening its assignment as a type II sulfatase (Fig. S2).

**Table 1:** Substrates screened for activity in the Rv3406 *in vitro* coupled assay.

Substrate	Activity
2-Ethylhexyl sulfate (2-EHS)	Yes
Pentyl sulfate	Yes
Hexyl sulfate	Yes
Heptyl sulfate	Yes
Taurine	No

We also demonstrated that Rv3406’s activity on 2-EHS is dependent on the cosubstrate αKG (Fig. 2A) and on the iron concentration in the buffer (Fig. 2B). AtsK from *P. putida* shows a significant rate enhancement with addition of ascorbate, presumably due to scavenging of reactive oxygen species that might otherwise inactivate the protein [11]. Similarly, Rv3406’s sulfatase activity was enhanced by ascorbate in the reaction buffer (Fig. 2C).

### Structure of Rv3406

**Figure 3:**
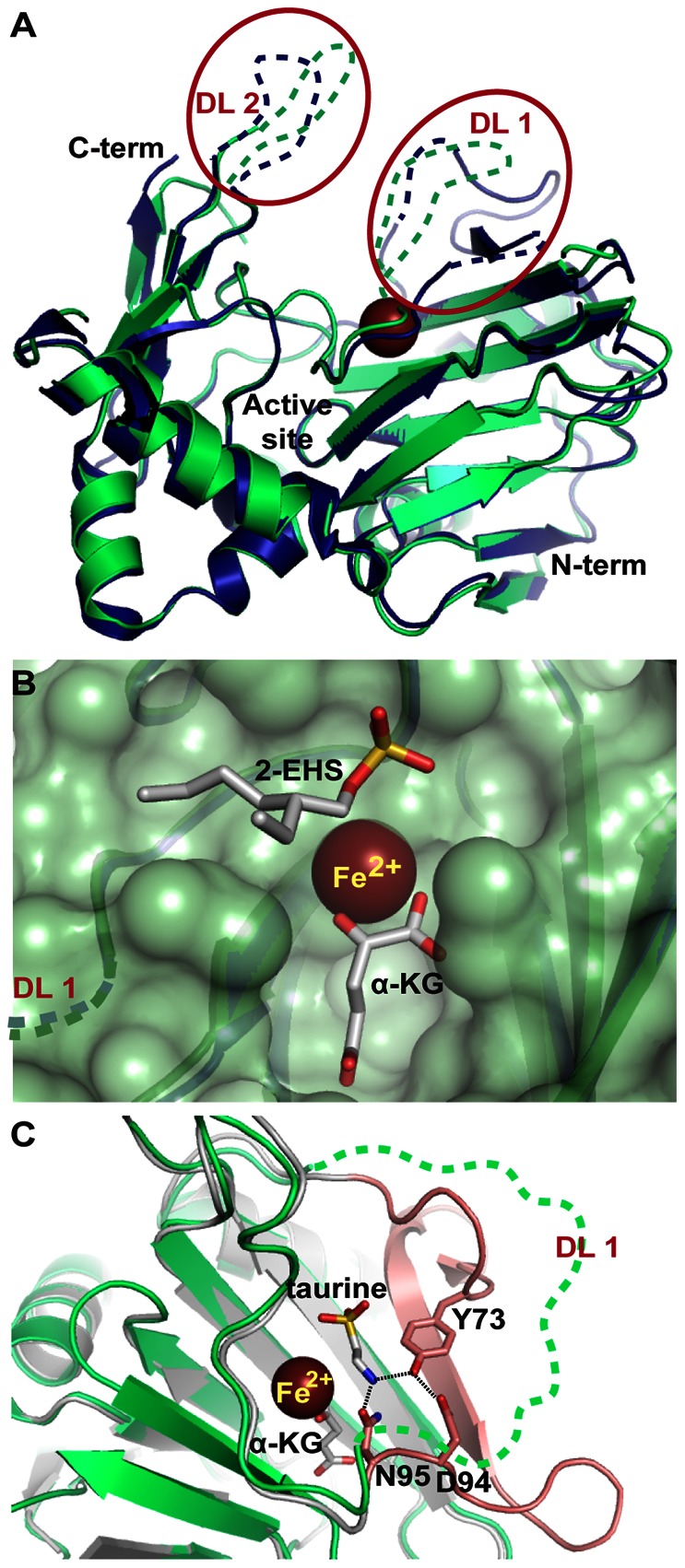
Analysis of the Rv3406 crystal structure. (A) Alignment of full length Rv3406 (green) with AtsK (PDB 1OIH, blue) with disordered loops modeled onto the structure. (B) A view of the active site with structures of the substrate and co-substrates modeled from AtsK. (C) Alignment of Rv3406 (green) with *E. coli* TauD (grey). Loop shown in pink contains the amino acids that hydrogen bond with taurine.

To provide additional support for a role for Rv3406 as a type II alkyl sulfatase, we obtained a crystal structure of the *apo* protein (2.5 Å, Table S1) (Fig. 3). Attempts to obtain diffraction-quality complexes via soaking and co-crystallization with alkyl sulfate substrates, iron, or cofactors proved unsuccessful. The *apo* crystal structure showed a near-perfect backbone alignment with the *apo* form of *P. putida* AtsK’s crystal structure [11] (Fig. 3A). Both structures contain the “jelly roll” arrangement of antiparallel beta strands present in other known non-heme iron and αKG-dependent dioxygenases (Fig. 3A). Rv3406 has two disordered loops adjacent to the active site that did not appear in the structure. The homologous loops in AtsK are also unstructured even in the co-crystals with substrate bound. The active sites of AtsK and Rv3406 contain the H-X-D/E-X_n_-H triad that comprises the iron binding site and are highly superimposable, including binding sites for 2-EHS and αKG. This allowed us to model co-substrates and alkyl sulfates into the active site of Rv3406 based on their empirically determined orientations in the AtsK structure (Fig. 3B) [11]. Due to the high degree of structural conservation between the active sites of Rv3406 and AtsK, we speculate that they operate via the same catalytic mechanism.

**Figure 4:**
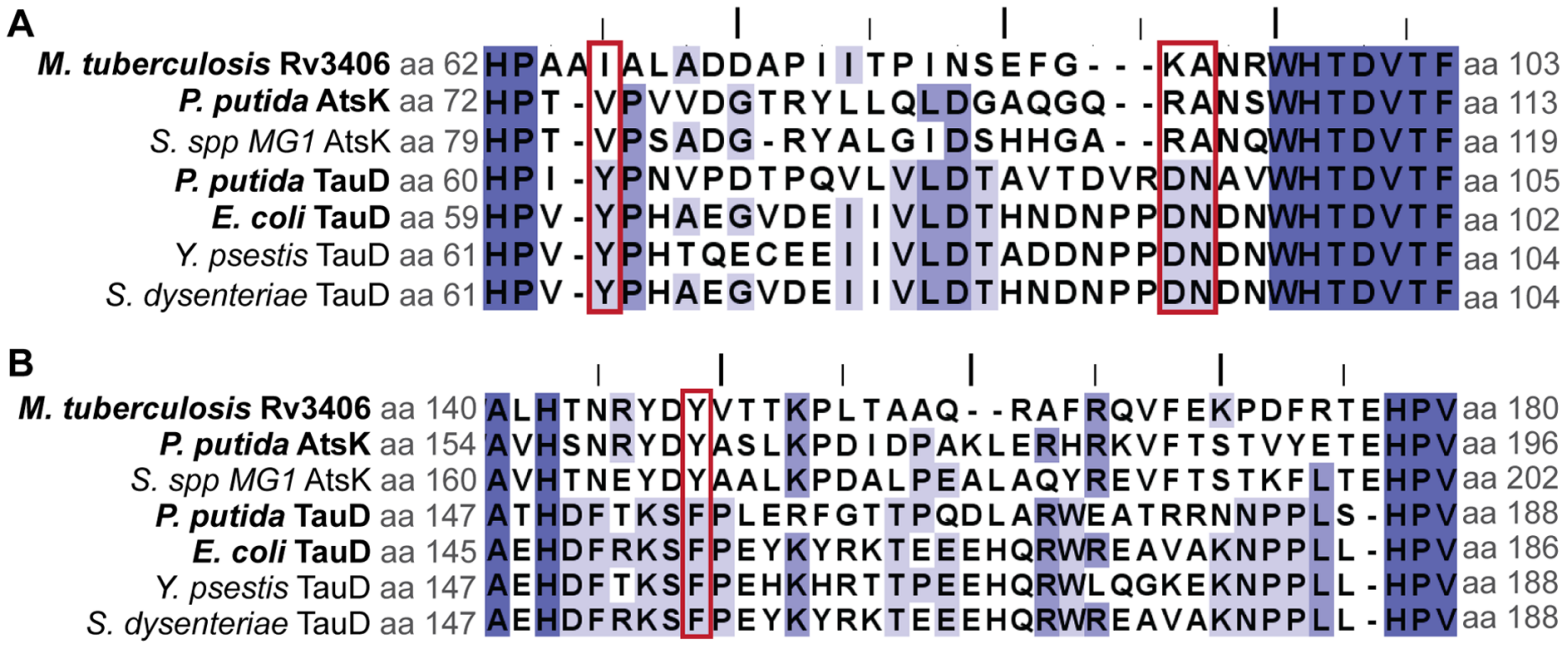
Protein alignment of alkyl sulfatase enzymes with taurine dioxygenase enzymes. **Enzymes in bold have been biochemically characterized.** (A) Alignment of disordered loop 1 where the red boxes are indicating the taurine binding residues in taurine dioxygenases and the analogous amino acids in alkyl sulfatase enzymes. (B) Alignment of disordered loop 2 where the red box is indicating the conserved phenylalanine in taurine dioxygenases and the analogous tyrosine in alkyl sulfate enzymes.

The non-heme iron and αKG-dependent dioxygenase superfamily also includes the well-studied taurine dioxygenases (TauD), which hydrolyze taurine to sulfite and aminoacetaldehyde. The type II sulfatases and TauD oxidize their substrates via similar mechanisms; accordingly, sequence alignment of Rv3406 and TauD indicates that the catalytic iron binding pockets are conserved (Fig. 3C). However, there are notable structural differences in the taurine binding region of TauD and the modeled 2-EHS binding region of Rv3406. Specifically, TauD enzymes contain conserved Tyr, Asn, and Asp residues (Y73, N95, and D94 in *E. coli* TauD) that form a hydrogen bonding network with the substrate’s amino group (Fig. 3C) [37]. Two of these taurine-binding residues are replaced by hydrophobic amino acids (Y73→Val or Ile, D94→Ala) in AtsK and Rv3406, and therefore would be unable to participate in hydrogen bonding with taurine (Fig. 4A). In Rv3406, the polar head group of the conserved Asn residue was oriented towards the solvent, creating a hydrophobic surface in the active site that might favor binding of a nonpolar alkyl sulfate substrate. As mentioned above, taurine was not a substrate for Rv3406 in the biochemical assay and, as well, did not inhibit Rv3406’s activity on 2-EHS (data not shown).

### Rv3406 Desulfates Alkyl Sulfate Substrates *in Vivo*

**Figure 5:**
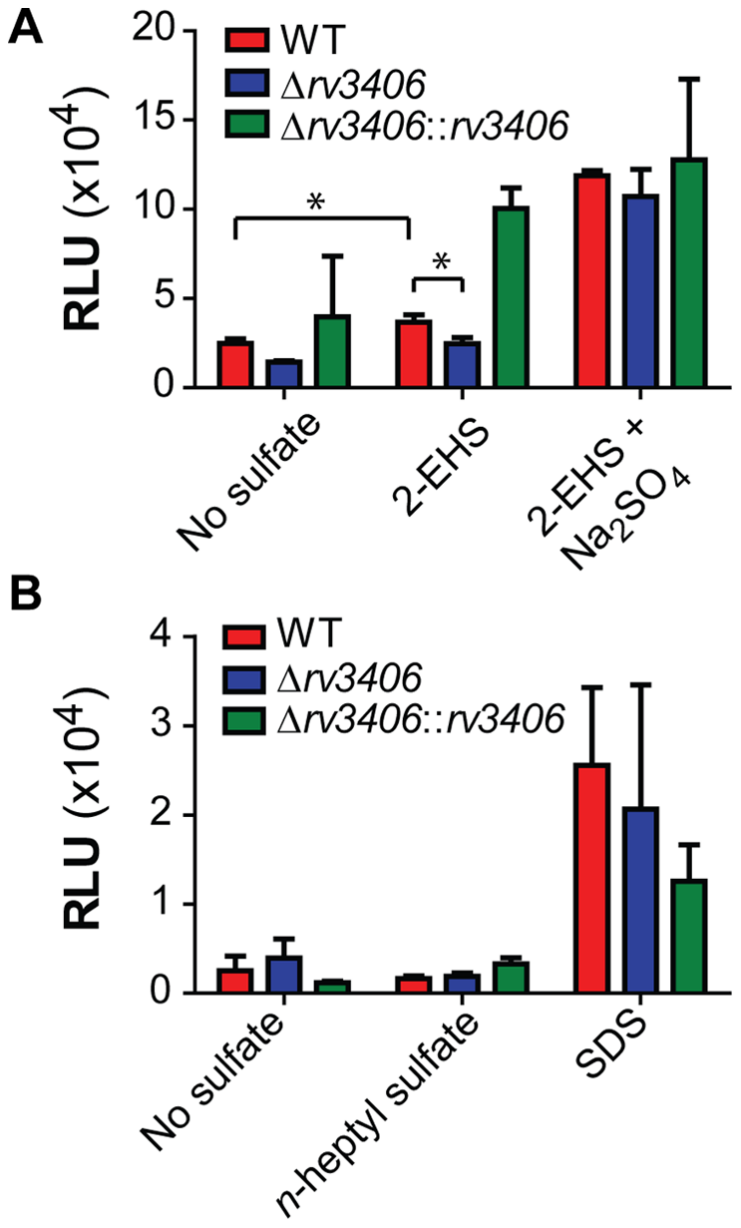
Rv3406 is essential in Mtb for growth on 2-EHS as the sole sulfur source. (A) Growth of Mtb strains using either 2-EHS alone or 2-EHS with sodium sulfate. (B) Growth of Mtb strains on *n*-heptyl sulfate or SDS. Data represents three biological replicates and error bars denote standard deviation. Asterisk indicates a p value of less than 0.005.

To investigate the function of Rv3406 *in vivo*, we generated an *rv3406* deletion (Δ*rv3406*) in the Mtb Erdman strain. We grew wild type (WT) Mtb, Δ*rv3406*, and the complemented strain (Δ*rv3406*::*rv3406*) in a chemically-defined Sauton media that lacked all sulfur or sulfate sources. We tested the ability of these strains to replicate using alkyl sulfate esters as their sole sulfur source. Both the WT and the complemented strain were able to grow in media containing 0.5 mM 2-EHS, while Δ*rv3406* showed no growth after 5 days (Fig. 5A). However, the complement showed greatly increased growth compared to WT. The Δ*rv3406* strain was complemented with *rv3406* under a strong constitutive promoter. As a result, the complemented strain most likely produces an abundance of Rv3406, which may lead to enhanced growth due to more efficient sulfur metabolism. In accordance with this proposal, we observed enhanced growth upon addition of sulfate esters to media (Fig. 5A). Unlike 2-EHS, *n*-heptyl sulfate was unable to support growth of any strain in sulfur-free media (Fig. 5B). It is possible that this substrate is not efficiently transported into the cell, where we presume Rv3406 resides.

Expression of Mtb *rv3406* was previously shown to be upregulated upon treatment of the bacteria with sodium dodecyl sulfate (SDS) [38]. Moreover, type II sulfatases in other organisms are active on SDS, thereby reducing its toxicity [39], [40]. Interestingly, all of our strains were able to replicate using SDS (0.02%) as a sole sulfur source. However, growth on SDS was not dependent on the presence of Rv3406.

## Discussion

Sulfate metabolism is important to the life cycle of Mtb infection [22]. Not only is sulfate involved in the biosynthesis of reduced sulfur metabolites such as methionine, cysteine and mycothiol [41], but it is also required for the production of cell surface sulfolipids that are thought to be involved in Mtb pathogenesis [42], [43], [44], [45], [46]. Mtb is highly adapted to scavenge essential nutrients, such as lipids as a carbon source [47] and iron [48], from the host. A sulfate scavenging mechanism may be analogously important for maintenance of sulfated and reduced sulfur-containing metabolites. Rv3406 is only the second alkyl sulfatase biochemically characterized from this family of non-heme iron-αKG dependent oxygenases. However, it remains to be determined whether sulfate scavenging by Rv3406 or other Mtb sulfatases is important for the pathogenesis of Mtb.

Substrate specificity and structural characterization of Rv3406 and its ortholog AtsK allowed us to compare the alkyl sulfatases to the homologous taurine dioxygenases (TauD). The *E. coli* TauD is one of the best studied enzymes in this family, with extensive characterization of catalytic intermediates and characterization of the substrate binding pockets [37], [49], [50]. Despite small alterations to the substrate binding pocket, the residues that enable iron, αKG and sulfate/sulfonate binding are conserved between TauDs and type II sulfatases. In TauD, McCusker and Klinman identified a phenylalanine residue (Phe159) that is essential for efficient substrate turnover. Positioned directly behind the bound taurine molecule, Phe159 holds the substrate within close proximity to the iron center and creates a lid over the active site [50]. Unfortunately, the analogous loop in Rv3406, containing Tyr149 in place of Phe159 (numbering from the Rv3406 structure, Fig. 4B), is disordered in the crystal structures of the alkyl sulfatases, even when substrate is bound [11]. Without structural information for this loop, it is difficult to predict the substrate recognition or catalytic consequences of replacing Phe159 with a Tyr residue.

Although both 2-EHS and *n*-heptyl sulfate were substrates for Rv3406 in our biochemical assay, only 2-EHS supported replication in otherwise sulfur-free media. This may reflect differential transport of these substrates to the cytosol, where we predict Rv3406 to be located based on its requirement for αKG, a cytosolic metabolite, as well as its lack of an obvious secretion signal and the high solubility of the recombinant protein expressed in *E. coli*. Furthermore, an earlier proteomics study indicated that Rv3406 may be associated with Mtb membrane components [51]; if so, its catalytic site is still likely to be cytosolic.

While we have identified 2-EHS as the best substrate for Rv3406, it is unlikely to be its physiological substrate. Rv3406 is the most conserved sulfatase across mycobacteria, from the soil dwelling species to the pathogenic species. It is also conserved in the highly reduced genome of *M. leprae*. The conservation of Rv3406 across a diverse array of mycobacterial species suggests a role in sulfate scavenging. Such a role could be satisfied by activity on a range of substrates available in the environment of a human host, making it difficult to speculate on the precise nature of Rv3406’s physiological substrates. Alternatively, Rv3406 could be involved in catabolism of an endogenous sulfated metabolite that has not yet been identified.

In conclusion, characterized the putative type II sulfatase Rv3406 from Mtb as a first step toward understanding its role in the Mtb lifecycle. We subjected Rv3406 to a variety of commercially available sulfate esters in an NADH/LADH coupled enzymatic assay. Rv3406 showed a similar substrate preference to its orthologue in *P. putida* AtsK, with 2-EHS being the best substrate and with straight chain alkyl sulfates exhibiting high activity. No activity was seen with carbohydrate substrates and only low activity observed with sulfated steroids. We also solved the crystal structure of the *apo* form of Rv3406. The overall structure aligns well with the AtsK structure and the two enzymes have highly superimposable active sites. Finally, we confirmed the activity of Rv3406 in Mtb cells by assessing whether a strain lacking *rv3406* (Δ*rv3406*) could replicate using alkyl sulfate esters as the sole sulfate source. Rv3406 was indeed essential for growth when 2-EHS was provided as the sole sulfate source. Rv3406 is only the second type II sulfatase characterized *in vitro* or *in vivo* and the first from a pathogenic organism. While the role of sulfatases in Mtb is still under investigation, our work represents an important step toward understanding how sulfatases affect the ability of Mtb to scavenge sulfur and persist as one of the world’s deadliest human pathogens.

## Supporting Information

Figure S1 (A) AtsK activity in coupled assay with 2-EHS. Red circles indicate assay with 1 mM 2-EHS, green squares are with 10 mM 2-EHS and blue triangles are a no enzyme control. (B) Rv3406 activity with two concentrations of 2-EHS and *n*-heptyl sulfate. All assays were done as described in the methods. Blue squares are 1 mM 2-EHS, blue triangles are 10 mM 2-EHS, and blue diamonds are a no enzyme control with 2-EHS. Red circles are 1 mM *n*-heptyl sulfate and red diamonds are a no enzyme control with *n-*heptyl sulfate. (C) Indicated the V_max_ of Rv3406 with *n*-pentylsulfate (blue), *n*-hexylsulfate (red), *n*-heptylsulfate (green) and 2-EHS (black). Rv3406 concentration was between 0.5 and 0.75 µM for all experiments. AtsK concentration was between 0.5 and 1 µM.
(TIF)

Figure S2 **Taurine is not a substrate for Rv3406.** Levels of sulfite were measured after incubation of Rv3406 or TauD with Taurine. Taurine in buffer was used as a negative control and samples were normalized to enzyme in their respective buffers. Rv3406 enzyme concentration was between 0.5 and 0.75 µM for all experiments. TauD concentration was 0.5 µM. *Values had negative absorbance.
(TIF)

Table S1 **Crystallographic Information.**
(DOC)

File S1 **Supportive materials and Methods.**
(DOC)
