# Supplementary figures and images for: Mycobacterium tuberculosis Rv3406 Is a Type II Alkyl Sulfatase Capable of Sulfate Scavenging
Source: PLoS One. 2013 Jun 6;8(6):e65080. doi: 10.1371/journal.pone.0065080 (PMC3675115; doi:10.1371/journal.pone.0065080)

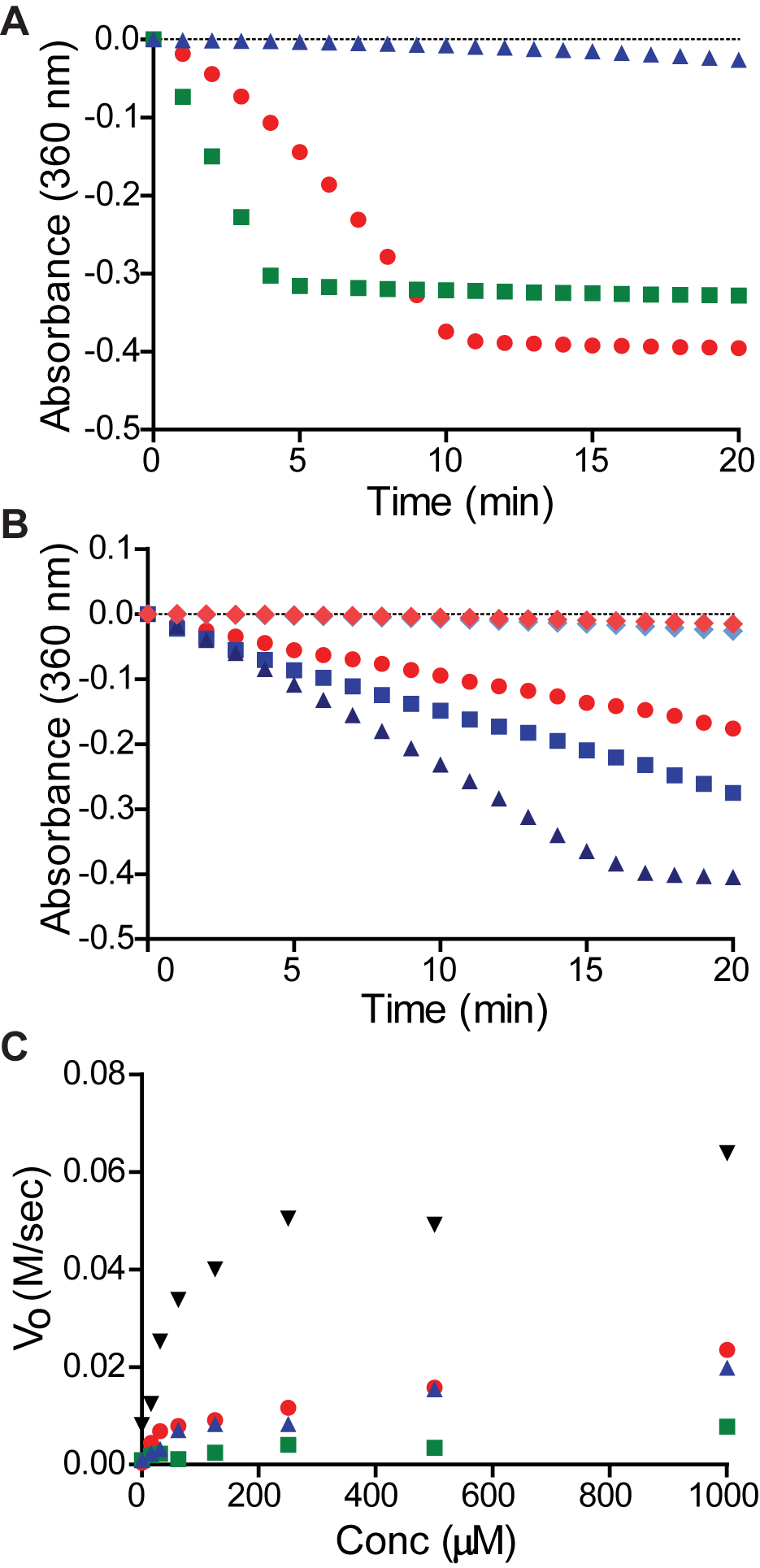

Supplement: Figure S1 — (A) AtsK activity in coupled assay with 2-EHS. Red circles indicate assay with 1 mM 2-EHS, green squares are with 10 mM 2-EHS and blue triangles are a no enzyme control. (B) Rv3406 activity with two concentrations of 2-EHS and n-heptyl sulfate. All assays were done as described in the methods. Blue squares are 1 mM 2-EHS, blue triangles are 10 mM 2-EHS, and blue diamonds are a no enzyme control with 2-EHS. Red circles are 1 mM n-heptyl sulfate and red diamonds are a no enzyme control with n-heptyl sulfate. (C) Indicated the Vmax of Rv3406 with n-pentylsulfate (blue), n-hexylsulfate (red), n-heptylsulfate (green) and 2-EHS (black). Rv3406 concentration was between 0.5 and 0.75 µM for all experiments. AtsK concentration was between 0.5 and 1 µM. (TIF) [file pone.0065080.s001.tif]

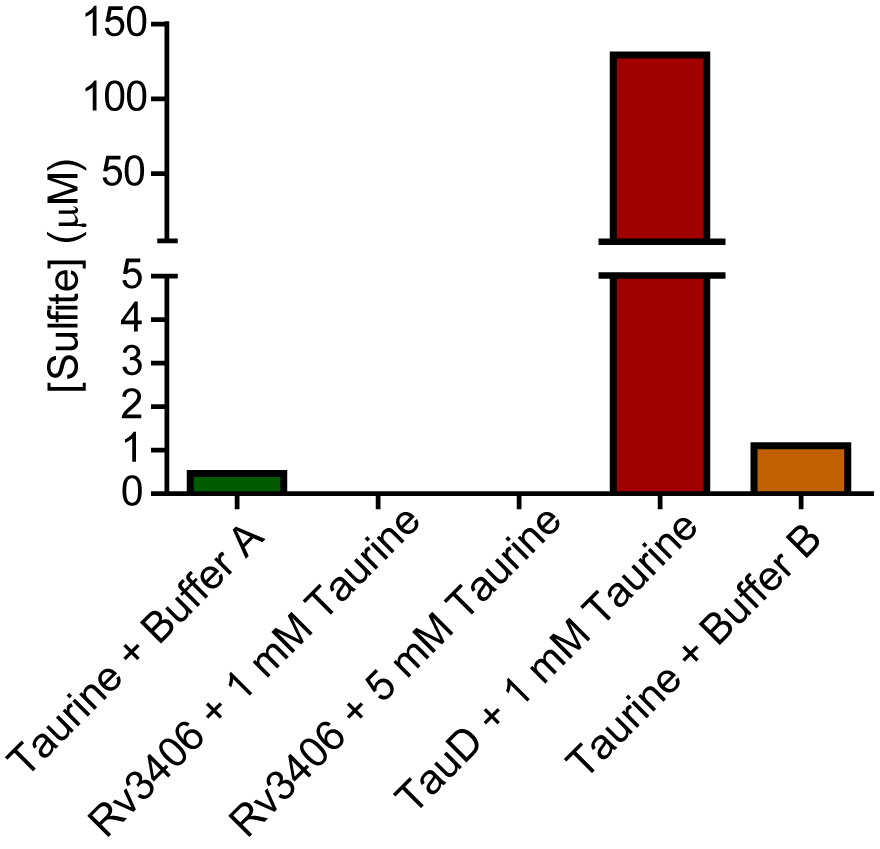

Supplement: Figure S2 — Taurine is not a substrate for Rv3406. Levels of sulfite were measured after incubation of Rv3406 or TauD with Taurine. Taurine in buffer was used as a negative control and samples were normalized to enzyme in their respective buffers. Rv3406 enzyme concentration was between 0.5 and 0.75 µM for all experiments. TauD concentration was 0.5 µM. *Values had negative absorbance. (TIF) [file pone.0065080.s002.tif]
