# Supplementary material for: Mycobacterium tuberculosis Rv3406 Is a Type II Alkyl Sulfatase Capable of Sulfate Scavenging
Source: PLoS One. 2013 Jun 6;8(6):e65080. doi: 10.1371/journal.pone.0065080 (PMC3675115; doi:10.1371/journal.pone.0065080)
Supplement: Table S1 — Crystallographic Information. (DOC) [file pone.0065080.s003.doc]

SI Table 1: Crystallographic Information

| **Data collection** | |
| --- | --- |
| Space group | P2_1_2_1_2_1_ |
| Unit cell axes (Å) | a=64.75, b=128.47, c=139.33 |
| Resolution limits (Å)(highest bin) | 45.60 – 2.50 (2.54 – 2.50) |
| Unique reflections | 40,338 |
| Average redundancy | 5.0 (5.6) |
| Average I / σ | 18.7 (4.2) |
| R_merge_ (%) | 7.3 (41.3) |
| Completeness (%) | 98.40 |

| **Refinement** | |
| --- | --- |
| R_work_/R_free_ (%) | 21.68 / 27.24 |
| No. protein molecules / ASU | 4 |
| No. protein residues | 946 |
| No. water molecules | 57 |
| Total atoms | 7490 |
| Rmsd bond angle deviations (°) | 1.06 |
| Rmsd bond length deviations (Å) | 0.007 |
| <B-factors> (Å^2^) | 67.40 |
| (ϕ,ψ)-favored / outliers (%) | 95.3 / 0.0 |
| PDB accession | 4FFA |

R_merge_ = Σ|I-<I>|/ΣI; I is reflection intensity

R_work_ = ΣF_obs_|-|F_calc_|| / Σ|F_obs_|; F_obs_ is observed structure-factor amplitude and F_calc_ is calculated structure factor amplitude. R_free_ is equivalent to R_work_ but is calculated for a randomly chosen 5% subset of reflections excluded from refinement.
