# Supplementary material for: Mycobacterium tuberculosis Rv3406 Is a Type II Alkyl Sulfatase Capable of Sulfate Scavenging
Source: PLoS One. 2013 Jun 6;8(6):e65080. doi: 10.1371/journal.pone.0065080 (PMC3675115; doi:10.1371/journal.pone.0065080)
Supplement: File S1 — Supportive materials and Methods. (DOC) [file pone.0065080.s004.doc]

**Supplemental information**

**Materials and methods**

*Rv3406 has no* in vitro *activity on taurine*

To test the activity of Rv3406 on Taurine, we used the standard assay conditions omitting the LADH and NADH (indicated as buffer A in SI Fig 2). Two concentrations of taurine were tested (1 mM and 5 mM). The experiments were done in 1 mL and were incubated at RT for 30 mins. The TauD positive control was done as described previously in ref . Briefly, the reaction contained buffer B (50 mM bis-Tris pH 6.2, 500 μM FeCl_2_, 10 mM αKG) and contained 1 mM Taurine. Reactions of 1 mL were initiated with addition of enzyme and incubated at RT for 30 mins. The reactions were quenched with 50 μL of 2.5 mM trichloroacetic acid followed by neutralization with 50 μL 2.5 M NaOH. Precipitates were removed by centrifugation and supernatants were transferred to a new tube. Sulfite concentration was determined using Ellman’s reagent. To quenched assay supernatants, 50 μL 5 mM Ellman’s reagent (5,5’-dithio-bis(2-nitrobenzoic acid) dissolved in 100 mM NaH_2_PO_4_ pH 7.2). The concentration of sulfite was determined by absorbance at 412 nm on a CARY 100 Bio UV-Visible Spectrophotometer with a range of 200-900 nm. Samples were normalized to a control reaction containing enzyme and no substrate. Concentrations were calculated from a sodium bisulfite standard curve.

**Supplemental figures**

**Figure S1:** (A) AtsK activity in coupled assay with 2-EHS. Red circles indicate assay with 1 mM 2-EHS, green squares are with 10 mM 2-EHS and blue triangles are a no enzyme control. (B) Rv3406 activity with two concentrations of 2-EHS and *n*-heptyl sulfate. All assays were done as described in the methods. Blue squares are 1 mM 2-EHS, blue triangles are 10 mM 2-EHS, and blue diamonds are a no enzyme control with 2-EHS. Red circles are 1 mM *n*-heptyl sulfate and red diamonds are a no enzyme control with *n-*heptyl sulfate. (C) Indicated the V_max_ of Rv3406 with *n*-pentylsulfate (blue), *n*-hexylsulfate (red), *n*-heptylsulfate (green) and 2-EHS (black).

**Figure S2:** Taurine is not a substrate for Rv3406. Levels of sulfite were measured after incubation of Rv3406 or TauD with Taurine. Taurine in buffer was used as a negative control and samples were normalized to enzyme in their respective buffers. *Values had negative absorbance.

**Reference**

1. McCusker KP, Klinman JP (2009) Modular behavior of tauD provides insight into the origin of specificity in alpha-ketoglutarate-dependent nonheme iron oxygenases. Proc Natl Acad Sci U S A 106: 19791-19795.
